# Northern Ireland farm-level management factors for prolonged bovine tuberculosis herd breakdowns

**DOI:** 10.1017/S0950268820002241

**Published:** 2020-09-28

**Authors:** L. P. Doyle, E. A. Courcier, A. W. Gordon, M. J. H. O'Hagan, P. Johnston, E. McAleese, J. R. Buchanan, J. A. Stegeman, F. D. Menzies

**Affiliations:** 1 Department of Agriculture, Environment and Rural Affairs, Veterinary Epidemiology Unit, Dundonald House, Upper Newtownards Road, Belfast BT4 3SB, Belfast, UK; 2 Statistical Services Branch, Agri-Food and Biosciences Institute, Newforge Lane, Belfast, BT9 5PX, UK; 3 Department of Agriculture, Environment and Rural Affairs, Veterinary Service Animal Health Group, Ballykelly House, 111 Ballykelly Road, Ballykelly, Limavady, BT49 9HP, Belfast, UK; 4 Department of Farm Animal Health, Faculty of Veterinary Medicine, University of Utrecht, Yalelaan 7, Utrecht, The Netherlands

**Keywords:** Bovine tuberculosis, case-control study, cattle, chronic breakdowns, epidemiology, *Mycobacterium bovis*

## Abstract

This study determined farm management factors associated with long-duration bovine tuberculosis (bTB) breakdowns disclosed in the period 23 May 2016 to 21 May 2018; a study area not previously subject to investigation in Northern Ireland. A farm-level epidemiological investigation (*n* = 2935) was completed when one or more Single Intradermal Comparative Cervical Test (SICCT) reactors or when one or more confirmed (positive histological and/or bacteriological result) lesion at routine slaughter were disclosed. A case-control study design was used to construct an explanatory set of management factors associated with long-duration bTB herd breakdowns; with a case (*n* = 191) defined as an investigation into a breakdown of 365 days or longer. Purchase of infected animal(s) had the strongest association as the most likely source of infection for long-duration bTB herd breakdowns followed by badgers and then cattle-to-cattle contiguous herd spread. However, 73.5% (95% CI 61.1–85.9%) of the herd type contributing to the purchase of infection source were defined as beef fattening herds. This result demonstrates two subpopulations of prolonged bTB breakdowns, the first being beef fattening herds with main source continuous purchase of infected animals and a second group of primary production herds (dairy, beef cows and mixed) with risk from multiple sources.

## Introduction

Bovine tuberculosis (bTB) caused by *Mycobacterium bovis* is a zoonotic disease primarily affecting animals. Although cattle are the main hosts, the disease has been reported in many other farmed and wild animals [1]. Department of Agriculture, Environment and Rural Affairs (DAERA) has a European Union (EU) Commission approved bTB eradication programme which ensures compliance with the EU Trade Directive 64/432/EEC. EU approval of the bTB Northern Ireland eradication programme is vital in safeguarding the export-dependent livestock and livestock products industry (worth in excess of £1.79 billion in 2018) [2]. In 2018, the Northern Ireland bTB programme cost £39 million, an increase of £8.5 million from 2016. This increase was reflective of increased disease incidence from 2016 to 2017 (herd incidence of 9.61% in December 2017 compared to 7.45% in December 2016) requiring associated increased expenditure largely in the area of compensation payment for the purchase of cattle as part of the bTB programme [3].

In 2016, a bTB eradication strategy for Northern Ireland was published [4], providing a framework for bTB eradication from the national cattle population. Part of the implementation plan for this strategy recommended that herds chronically infected with bTB (‘chronic herds’) should be recognised as a distinct entity for action and a package of measures be targeted at them so as to minimise their impact.

In a previous publication [5], data from a national database Animal and Public Health Information System (APHIS) [6] were used to determine definitions for chronic herds. The definitions developed for both long-duration and recurrent bTB herd breakdowns encompassed almost 40% of the total number of Single Intradermal Comparative Tuberculin Test (SICCT) reactors identified during the study period [5]. This study looked at risk factors pertaining to the SICCT and cattle movement data stored on APHIS, implementing a design (same design as our present study) which compared prolonged bTB breakdowns to short-duration breakdowns [5]. However, the original study [5] could not investigate any of the bTB herd breakdown risk factors associated with chronic herds at a farm management level, a knowledge gap in Northern Ireland which our present study aimed to fill. Such management factors have been studied in Great Britain and the Republic of Ireland [7, 8]. A previous case-control study in Northern Ireland considered risk factors for bTB relating to farm boundaries, neighbouring herds and wildlife, but it did not investigate chronic bTB herd breakdowns [9] and in a design contrast to our study it compared herds with breakdowns to herds which did not experience bTB breakdowns.

### Study objective

The objective of this study was to identify farm-level management factors associated with prolonged bTB herd breakdowns, using data collected during on-farm epidemiological investigations. A case-control study design was used where prolonged duration bTB herd breakdowns (as defined previously [5]) were compared to short-duration bTB herd breakdowns.

## Materials and methods

### Study design and data collection

**Fig. 1.**
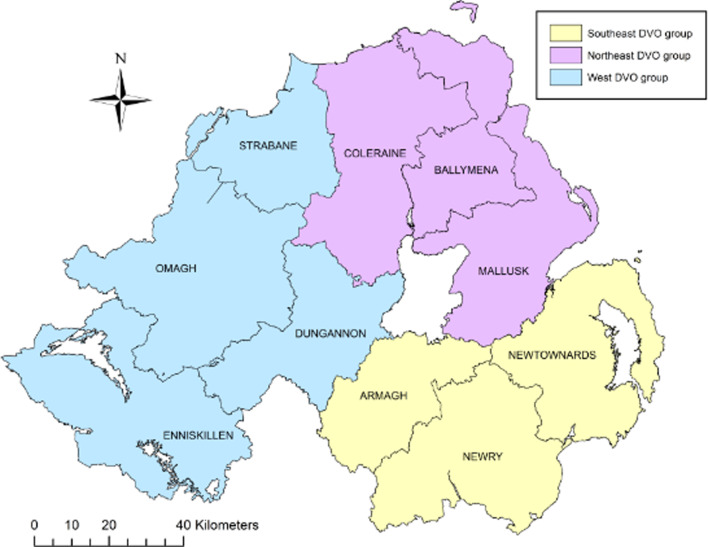
Northern Ireland DVOs aggregated into three groups, southeast, northeast and west.

A case-control study was conducted on a study population consisting of all bTB herd breakdown investigations during the period 23 May 2016 to 21 May 2018. Data collection involved completion of an on-farm investigation form when one or more SICCT reactors or one or more confirmed lesion at routine slaughter (LRS) were disclosed in any Northern Ireland cattle herd. Confirmation of bTB in an LRS was defined as a positive histological and/or bacteriological culture result following laboratory examination. Investigations were carried out by trained Animal Health and Welfare Inspectors (AHWI) who visited each of the bTB breakdown farms. At each farm, an on-site questionnaire was completed (Supplementary Table S2) through face-to-face interview of the farmer, including identification of all herds contiguous to the bTB herd breakdown. Based on the completed questionnaire and local knowledge of the area, the Veterinary Officer (VO) responsible for the bTB herd breakdown, where possible, determined the most likely source of infection for the breakdown. Questionnaire information along with data extracted from APHIS (herd size and location) was collated into Microsoft Access^TM^ (Microsoft Corporation, Redmond, WA, USA). For our study, the 10 Divisional Veterinary Offices (DVOs) were aggregated into three groups according to their geographic location: south east group (Armagh, Newry, Newtownards), west group (Dungannon, Enniskillen, Strabane, Omagh) and north east group (Ballymena, Coleraine, Mallusk) (Fig. 1).

Case and control definitions were identical to those used in a previous study [5]. Cases were bTB herd breakdowns which ended during the study period (23 May 2016 to 21 May 2018) and had a duration of ≥365 days. Controls were bTB herd breakdowns which ended during the study period (23 May 2016 to 21 May 2018) and had a duration of <365 days.

### Data analysis

Microsoft Access^TM^ (Microsoft Corporation) and R Version 3.4.0^1^ were used for data manipulations and R Version 3.4.0^a^ and Stata/SE 15^2^ were used for data analysis. The model framework used was binary logistic regression using a purposeful selection of covariates [10] with the case definition forming the response variable. In total, 78 explanatory variables were derived from the on-farm questionnaire (see Supplementary Table S2) along with their associated factor levels. Initially, all variables were tabulated using the duration case definition against each variable's factor levels. As variables were added or removed from the model, the Akaike Information Criterion (AIC) difference was calculated between the old and new proposed model, in order to determine if the proposal reduced AIC by a value greater than two [10]. Where the models were subsets of each other, the Likelihood Ratio test (LRT) was also calculated in order to determine if addition or removal of variables was significant at the *P* ≤ 0.05 level.

Initial analysis was by univariable logistic regression. Any variables containing low numbers (<10) of cases at any factor level, which could not have that factor level logically merged with another level were removed after univariable analysis. Remaining variables with *P* ≤ 0.25 were then analysed using a multivariable logistic regression model. The resultant model was further refined to produce a reduced multivariable logistic regression model which utilised variables with *P* ≤ 0.05 from the first multivariable model. Following the fit of the reduced multivariable model, its estimated coefficients were compared to those in the initial multivariable model to determine if there was a magnitude change of >20%. This magnitude change known as 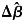
% (Delta-Beta-Hat %) indicates that one or more of the excluded variables are important in the sense of providing a needed adjustment effect of the variables that remained in the model [10]. Variables which formed the first multivariable model but not included in the initial reduced multivariable model were added back individually; being retained if they contributed to the overall model and reduced 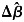
% to below 20%.

Further to this, variables with *P* > 0.25 in the initial univariable analysis were also individually added back in to determine if they contributed to the multivariable model thus producing the preliminary main effects model. The only continuous variable included in the preliminary main effects model was herd size. Fractional polynomial analysis [11] was applied to herd size in order to determine if it required scale transformation so as to satisfy the assumption of linearity in the logit outcome. Completion of this stage produced the main effects model. Using the variables present in the main effects model, all combinations of two-way interactions were statistically assessed using the LRT (*P* < 0.05); however, only those with probable clinical significance were accepted as potential candidates for the model. Interaction terms accepted into the final model had an odds ratio calculated as a linear combination with their associated main effects (*β*_0_ + *β*_1_ + *β*_2_ + *β*_1_.*β*_2_) and the results placed into Table 1. The finalised model was then subjected to the Hosmer–Lemeshow goodness-of-fit-test (decile sub-grouped) to determine how well it fitted the data. Table 2 details how the methodology was applied in this study to achieve the final multivariable model.

**Table 1.** Results of final multivariable case-control study containing calculated effects for the two-way interactions included in the model

Variable interactions		Odds ratio	95% CI
Mixed grazing of cattle and sheep	Do cattle drink from natural sources of water		
Yes	No	1	–
Yes	Yes	0.651	0.286–1.481
Divisional Veterinary Office of the bTB breakdown	Any woodland on the farm or within 1.6 km of the farm		
Armagh, Newry, Newtownards (South East)	No	1	–
	Yes	0.520	0.201–1.207
Dungannon, Enniskillen, Strabane, Omagh (West)	No	1	–
	Yes	0.492	0.205–1.252
Ballymena, Coleraine, Mallusk (North East)	No	1	–
	Yes	0.453	0.161–1.271
Divisional Veterinary Office of the bTB breakdown (Fig. 2)	Herd size transformed (increase in value by one)		
Armagh, Newry, Newtownards (South East)	No	1	–
	Yes	0.968	0.892–1.043
Dungannon, Enniskillen, Strabane, Omagh (West)	No	1	–
	Yes	1.005	0.907–1.132
Ballymena, Coleraine, Mallusk (North East)	No	1	–
	Yes	1.069	0.899–1.124
bTB breakdown risk picked as the most likely source by VO (Fig. 3)	Herd size transformed (increase in value by one)		
Source of infection not established (includes Other and Deer source)	No	1	–
	Yes	0.968	0.892–1.043
Cattle to cattle contiguous herd spread	No	1	–
	Yes	1.046	0.993–1.173
Purchase of infected animal(s)	No	1	–
	Yes	1.119	0.993–1.261
Carryover of previous infection	No	1	–
	Yes	1.002	0.865–1.161
Badgers	No	1	–
	Yes	1.071	0.954–1.203
Purchase of store cattle at a market within the previous 5 years (Fig. 4)	Herd size transformed (increase in value by one)		
Yes	No	1	–
Yes	Yes	1.050	0.950–1.160

**Table 2.** Table showing methods applied and results observed at each stage of the study model building process

Stage	Study methods	Study results
1	Univariable logistic regression applied to the 78 variables initially derived from farm questionnaire	Odds ratio and associated *P*-value calculated for each level of all 78 variables
2	Each of the 78 variables derived from the questionnaire was tabulated at each of its factor levels to determine the number of cases/controls present at that factor level	11 variables were removed where factor levels contained <10 cases and could not be logically merged with another level. As a result this left 67 variables to carry forward for multivariable analysis
3	Multivariable logistic regression applied to all variables with *P* ≤ 0.25 from stage 1 and not removed at stage 2	Multivariable model containing 47 variables selected from stage 1 at the *P* ≤ 0.25 level and not removed at stage 2 (20 of the 67 variables had *P* > 0.25 and not reintroduced until stage 8)
4	Reduced multivariable model generated from variables with *P* ≤ 0.05 outputted from stage 3	Reduced multivariable model containing 11 variables selected from stage 3 at the *P* ≤ 0.05 level (36 of the 47 variables had *P* > 0.05)
5	Calculation of 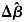 % (<20%) for reduced model produced at stage 4	The variable VO choice of the most likely source of infection had 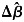 % >20%, thus some of the variables removed at stage 4 should be reassigned to model
6	Individual reassignment of variables removed at stage 4 to the reduced model to determine if these variables contributed to the overall model	Each of the 36 variables removed at stage 4 added back individually to determine if they contribute to overall model (LRT at *P* ≤ 0.05)
7	Reduced multivariable model refined by variable addition/removal to obtain 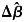 % <20% for all variables included in the multivariable model.	Refining of reduced multivariable model from stage 4 results in addition to four new variables: (1) Purchase of store cattle <5 years previous. (2) Manure spread on grazing ground. (3) VO second choice source of infection (4) Herd vaccinated against IBR. All 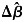 % for variables in the multivariable model at this stage of model building were <20%
8	Individual reassignment of variables removed at stage 3 to the model produced at stage 7 to determine if these variables contributed to the overall model. Output from this stage formed the preliminary main effects model	Each of the 20 variables removed at stage 3 added back individually to determine if they contribute to the overall model (LRT at *P* ≤ 0.05). Addition of the variable – herd vaccination against Leptospirosis gives the preliminary main effects model
9	Fractional polynomial analysis to assess the linearity of any continuous variables to the outcome	Analysis results in the variable transformation of herd size to a power of 0.5. Herd size is added in its transformed state to the preliminary model to form the main effects model and was referred to as herd size transformed
10	All combinations of two-way interactions (LRT at *P* ≤ 0.05 and judged as clinically significant) from main effects model were added to main effects model. Interactions retained or removed based on *P*-value (*P* ≤ 0.05) within the model and as to whether they lead to model improvement (AIC and LRT cut-offs already described). These interaction variable pairs were then evaluated as a linear combination (*β*_0_ + *β*_1_ + *β*_2_ + *β*_1_.*β*_2_) with the main effects to determine their overall odds ratio and associated confidence interval (Table 1)	Five interaction terms were added to the main effects model of 16 variables to form the final model. Odds ratio for these interactions in linear combination with their main effects were calculated and added to Table 1 (1) Mixed grazing of cattle/sheep × Cattle drink from natural sources of water (LRT: *P* = 0.015) (2) Any woodland on farm or <1.6 km of the farm × DVO of the bTB breakdown (LRT: *P* = 0.017) (3) DVO of the bTB breakdown × Herd size transformed (LRT: *P* = 0.020) (4) bTB breakdown risk most likely source chosen by VO × Herd size transformed (LRT: *P* = 0.001) (5) Purchase of store cattle at market <5 years × Herd size transformed (LRT: *P* = 0.001)
11	Application of the goodness of fit test (Hosmer and Lemeshow goodness of fit test) and variable correlation analysis to the final model	Hosmer and Lemeshow goodness of fit test result: *P* = 0.582. Provides evidence at *P* ≤ 0.05 level of adequate goodness of fit

## Results

A total of 2935 bTB herd breakdown investigations were completed during the study period. Supplementary Table S1 provides summary details of the 78 study variables for cases and controls. There were 126 ongoing bTB herd breakdowns at the end of the study period which were removed, leaving 2809 valid bTB herd breakdowns (191 cases and 2618 controls; Supplementary Table S1). Table 2 details the results returned at each stage of the model building process from univariable analysis through to final multivariable model. As a result of carrying out fractional polynomial analysis [11] on herd size, it was transformed to herd size to power 0.5 and was then referred to as ‘herd size transformed’ in the subsequent analysis.

The results from the final model (Table 3) demonstrated that the odds ratio of a bTB herd breakdown persisting >365 days that contained pedigree animals was 0.594 (95% CI 0.402–0.863); where fluke treatment was carried out on the farm was 0.263 (95% CI 0.139–0.528), where cattle can access grazing ground to which slurry has been freshly applied was 0.525 (95% CI 0.283–0.915); where there was partial upgrading of boundary fences in the last 3 years was 0.383 (95% CI 0.247–0.588); where there was a full upgrade of boundary fences in the last 3 years was 0.599 (95% CI 0.406–0.886); where dead badgers were found on roads within 1.6 km of the farm in the past 3 years was 1.810 (95% CI 1.268–2.616); manure spread on grazing ground was 1.289 (95% CI 0.926–1.798); use of IBR vaccination on the farm was 1.476 (95% CI 1.005–2.158) and use of leptospirosis vaccination on the farm was 0.631 (95% CI 0.391–0.999). Dairy herds accounted for 73.2% (95% CI 59.6–86.7%) of bTB herd breakdown which carry out leptospirosis vaccination. The presence of a badger sett was recorded on 29.87% (95% CI 28.18–31.56) of investigations and of these 2.95% (95% CI 2.33–3.58%) reported fencing off badger setts and latrines.

**Table 3.** Results of final multivariable case-control study containing categorical and continuous variables (note interaction terms are included in Table 1)

Study variable categorical	Exposure level	Case: TB breakdown with duration 365 days or greater (*n* = 191)		Control: TB breakdown with duration less than 365 days (*n* = 2618)		Odds ratio	95% CI	*P*-value
	(NA = Not Applicable)	*n*	%	*n*	%			
Registered pedigree animals present on the farm	No	144	7.6	1751	92.4	–	–	–
	Yes	47	5.1	867	94.9	0.594	0.402–0.863	0.007
Mixed grazing of cattle and sheep	No	139	6.3	2074	93.7	–	–	–
	Yes	52	8.7	544	91.3	2.278	1.328–3.810	0.002
Do cattle drink from natural sources of water	No	95	6.2	1443	93.8	–	–	–
	Yes	96	7.6	1175	92.4	1.655	1.136–2.416	0.009
Fluke treatment carried out on the farm	No	15	17.4	71	82.6	–	–	–
	Yes	176	6.5	2547	93.5	0.263	0.139–0.528	0.000
Cattle access to grazing ground to which slurry freshly applied	No or NA	174	7.1	2289	92.9	–	–	
	Yes	17	4.9	329	95.1	0.525	0.283–0.915	0.031
Any boundary fence with a neighbour upgraded in the past 3 years (where full upgrade is installation of a complete new fence)	No	63	9.2	621	90.8	–	–	–
	Some upgrading	43	4.2	982	95.8	0.383	0.247–0.588	0.000
	Full upgrading	85	7.7	1015	92.3	0.599	0.406–0.886	0.010
Any dead badgers seen on roads within 1.6 km from any of your land in past 3 years	No	181	6.9	2462	93.1	–	–	–
	Yes	10	6.0	156	94.0	1.81	1.268–2.616	0.001
Any woodland on the farm or within 1.6 km of the farm	No	93	7.8	1095	92.2	–	–	–
	Yes	98	6.1	1523	93.9	0.524	0.310–0.878	0.015
Divisional Veterinary Office of the bTB breakdown	Armagh, Newry, Newtownards	73	9.1	733	90.9	–	–	–
	Dungannon, Enniskillen, Strabane, Omagh	76	5.7	1252	94.3	0.383	0.161–0.910	0.030
	Ballymena, Coleraine, Mallusk	42	6.2	633	93.8	0.071	0.018–0.247	0.000
bTB breakdown risk picked as the most likely source by VO	Source of infection not established (includes Other and Deer source)	38	4.3	844	95.7	–	–	–
	Cattle to cattle contiguous herd spread	44	7.0	589	93.0	0.495	0.166–1.447	0.203
	Purchase of infected animal(s)	49	9.9	448	90.1	0.328	0.103–1.018	0.055
	Carryover of previous infection	13	6.6	184	93.4	0.896	0.150–4.773	0.901
	Badgers	47	7.8	553	92.2	0.573	0.189–1.703	0.320
Purchase of store cattle at a market within the previous 5 years	No	90	5.5	1545	94.5	–	–	–
	Yes	101	8.6	1073	91.4	0.604	0.271–1.333	0.215
Manure spread on grazing ground	No	88	5.9	1410	94.1	–	–	–
	Yes	103	7.9	1208	92.1	1.289	0.926–1.798	0.133
bTB breakdown risk picked as the second most likely source by VO	Source of infection not established (includes Other and Deer source)	17	6.0	267	94.0	–	–	–
	Cattle to cattle contiguous herd spread	45	9.0	454	91.0	1.181	0.627–2.312	0.616
	Purchase of infected animal(s)	13	8.6	138	91.4	1.481	0.638–3.385	0.353
	Carryover of previous infection	12	9.5	114	90.5	1.294	0.540–3.021	0.554
	Badgers	47	7.5	577	92.5	1.503	0.778–3.022	0.237
	No second choice source selected	57	5.1	1068	94.9	0.709	0.379–1.383	0.297
Herd vaccinated against IBR	No	111	5.6	1853	94.3	–	–	–
	Yes	80	9.5	765	90.5	1.476	1.005–2.158	0.045
Herd vaccinated against leptospirosis	No	174	6.7	2439	93.3	–	–	–
	Yes	17	8.7	179	91.3	0.631	0.391–0.999	0.054
Study variable continuous	Exposure level	Mean (95% CI)				Odds ratio	95% CI	*P*-Value
		Case		Control				
Herd size transformed (case/control exposure levels refer to herd size)	Average herd size as number of animals over the time period 2016–2018	210.90 (180.48–242.32)		129.83 (124.60–135.06)		0.968	0.892–1.043	0.41

**Fig. 2.**
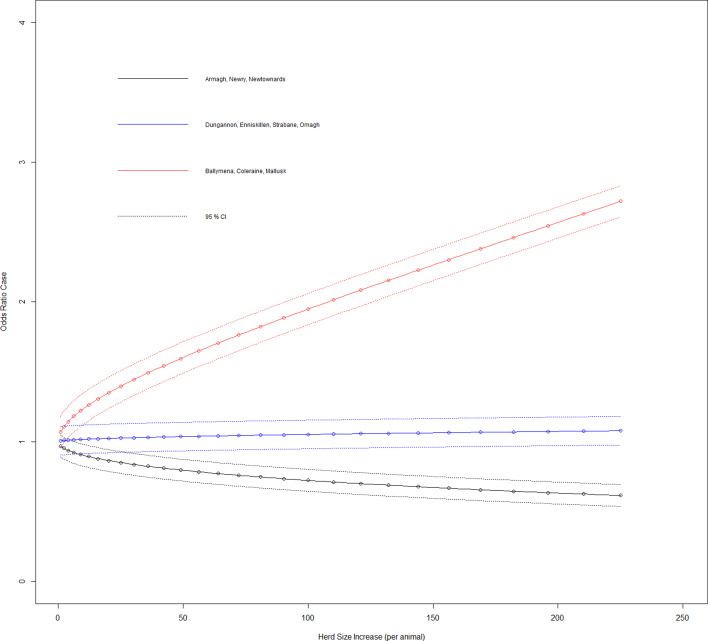
Duration case odds ratio for DVO of herd given effect of increasing herd size (variable herd size graphed in untransformed state).

**Fig. 3.**
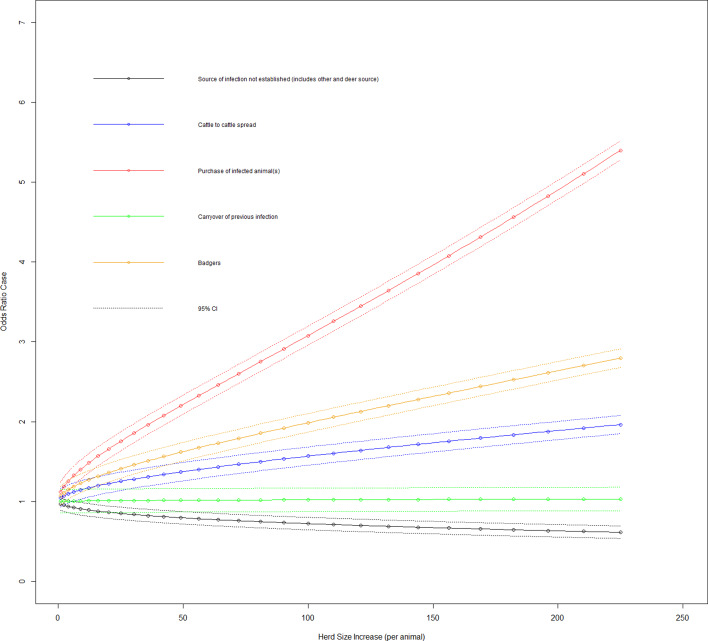
Duration case odds ratio for bTB breakdown by the most likely VO source given the effect of increasing herd size (variable herd size graphed in untransformed state).

**Fig. 4.**
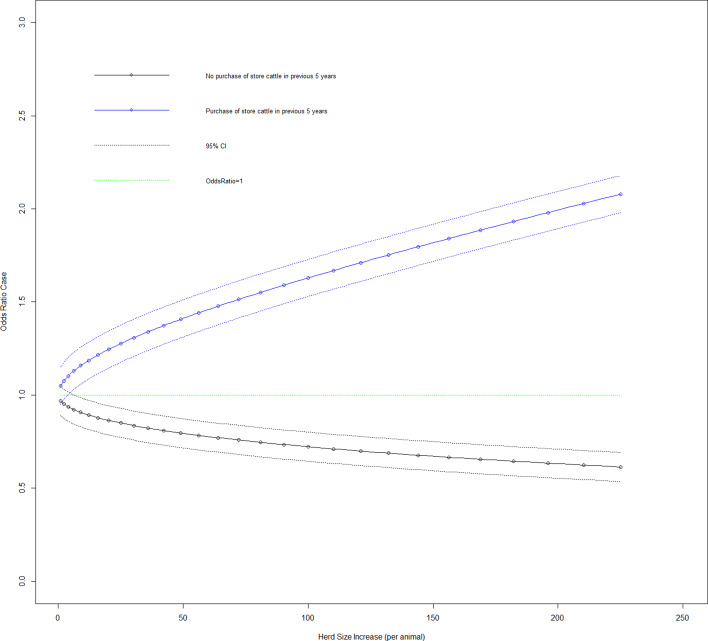
Duration case odds ratio for the purchase of store cattle in the previous 5 years given the effect of increasing herd size (variable herd size graphed in untransformed state).

The results of the linear combination of the five two-way interaction terms added to the model and their associated main effects (*β*_0_ + *β*_1_ + *β*_2_ + *β*_1_.*β*_2_) are shown in Table 1. Of the five two-way interaction terms added to the main effects model (Table 1), ‘mixed grazing of cattle and sheep’ × ‘do cattle drink from natural sources of water’ and ‘DVO of the bTB breakdown’ × ‘woodland on the farm or within 1.6 km of the farm’ returned odds ratios not significantly different from one, when interpreted as a linear combination with the main effects.

With the other three interaction terms, ‘DVO of the bTB breakdown’ × ‘herd size transformed’, ‘bTB breakdown risk picked as the most likely source by VO’ × ‘herd size transformed’ and ‘purchase of store cattle at a market within previous five years’ × ‘herd size transformed’ have their results from Table 1 shown in Figures 2–4, respectively, so that these can be interpreted in association with increasing herd size (untransformed). The odds ratio of a case where a farm is located in DVO south east group and there is an increase in herd size transformed by one was 0.968 (95% CI 0.895–1.047), with DVO west group and herd size transformed increase by one was 1.005 (95% CI 0.907–1.132) and with DVO north east group and herd size transformed increase by one was 1.069 (95% CI 0.899–1.124).

Figure 2 shows the effect on the duration case definition as herd size increases in each of the three DVO groups. The variables most likely risk source and herd size transformed had a significant interaction on addition to the main effects model (LRT: *P* = 0.001). The odds ratio of a case for the baseline group and where there is an increase in herd size transformed by one had was 0.968 (95% CI 0.895–1.047), with cattle to cattle contiguous herd spread of infection source and herd size transformed increase by one was 1.046 (95% CI 0.993–1.173), with purchase of infected animal(s) source and herd size transformed increase by one was 1.119 (95% CI 0.993–1.261), with a carryover of infection source and herd size transformed increase by one was 1.002 (95% CI 0.865–1.161) and with badger infection source and herd size transformed increase by one was 1.071 (95% CI 0.954–1.203).

Figure 3 shows the effect on the duration case definition of increasing herd size in each of the five infection source groups. Looking at herd types for the duration case definition based on whether they are beef fattening or other (dairy, beef cow or mixed), 11.4% (95% CI 1.9–20.7%) of beef fattening herds have source of infection as cattle to cattle spread, 73.5% (95% CI 61.1–85.9%) have purchase of infection as source, 0% for carryover of infection and 12.8% (95% CI 3.2–22.3%) have a badger source of infection. Variables purchase of store cattle at a market in the previous 5 years and herd size transformed had a significant interaction on addition to the main effects model (LRT: *P* < 0.001). The odds ratio of a case where a farm purchases store cattle from a cattle market, there was an increase in herd size transformed by one was 1.050 (95% CI 0.950–1.160). Figure 4 shows the effect on the duration case definition of increasing herd size for purchase of store cattle *vs.* non-purchase.

## Discussion

One of the key perspectives of this work is to further elucidate chronic long-duration bTB herd breakdowns (>365 days) through the provision of a quantitative characterisation of their infection source thus facilitating the formulation of a more focused disease control policy. Cattle movement has been identified as a consistent herd-level risk factor for bTB [12]. In our study, two cattle movement factors were statistically associated with long-duration bTB herd breakdowns, namely, purchase of cattle generally and specifically purchase of store cattle (calves purchased for feeding over winter). Both of these factors had a significant interaction with herd size (Fig. 4), which demonstrated that given purchase of cattle risk factors, larger herds were more likely to have prolonged bTB herd breakdowns.

Cattle purchase had the strongest association as the most likely infection source (Fig. 3) followed by wildlife (badgers) and then contiguous herd spread (cattle to cattle). However, when selectively looking at prolonged bTB herd breakdowns, three-quarters (73.5%) of the herd type contributing to the purchase of infection source were beef fattening herds. This highlights two subpopulations of long-duration bTB herd breakdowns, the first being beef fattening herds which have long-duration breakdowns due to continuous purchase of infected animals and a second group of primary production herds (dairy, beef cows and mixed) with long-duration breakdowns due to infection risk from multiple sources.

After purchase of infection, badgers formed the next most likely source of a long-duration bTB herd breakdown, which was a risk factor for bTB breakdowns identified in other studies [13–16]. Skuce *et al*. generalise the risk from badgers to indicators of badger density/activity [12] and, in terms of chronic bTB herd breakdowns in Republic of Ireland (ROI), badger presence was reported as a risk factor for dairy herds [7]. The findings from our study are consistent with these previous studies, with the association with prolonged bTB herd breakdowns being further affirmed by the finding of dead badgers on a road <1.6 kms from the home farm.

A VO attributing badgers as the most likely infection source also had significant interaction with herd size (Fig. 3) which showed this source in larger herds increased the odds of a prolonged bTB herd breakdown. This is not surprising as large herds tend to require a larger grassland area for feeding purposes, which increases the probability of exposure to a larger number of badgers, which is compounded by the larger number of cattle in such herds.

Two badger-related variables excluded from the model due to low case numbers were fencing off of badger setts and fencing off of badger latrines; both of which are considered preventive measures. An important observation in this study was that almost one-third (30%) of farms were observed as having badger setts, but only 3% of investigations reported farms where badger setts and/or latrines were fenced off, an observation also reported in other Northern Irish work [14]. Without extensive fencing off of badger setts and latrines indirect contact between badgers and cattle cannot be curtailed [17].

The third important source of infection was cattle-to-cattle contiguous herd spread. Previously, Denny and Wilesmith reported that in Northern Ireland, the two main associations with bTB breakdowns were the presence of badgers and contiguous neighbours who had confirmed bTB breakdowns (aetiological fraction for both was approximately 40% each) [9]. They also stated that 79% of fences in Northern Ireland did not prevent nose to nose contact between herds [9]. In a more recent Northern Ireland study, contact between neighbouring cattle was assessed as possible through 66.8% of boundaries, however no significant association was found between boundary contact and bTB breakdown [14]. Our study, which looked specifically at prolonged bTB herd breakdowns, found that both recent upgrading or complete installation of new boundary fences showed a significant negative association with the duration of bTB breakdowns (OR 0.383; 95% CI 0.247–0.588 and OR 0.599; 95% CI 0.406–0.886, respectively). This result provides circumstantial evidence for the application of better biosecurity measures in the form of adequate boundary fences to reduce cattle-to-cattle contiguous herd contact could reduce the odds of a prolonged bTB herd breakdown.

This study also investigated associations with other common diseases found on Northern Ireland farms. Application of fluke treatment was significantly associated with a reduced odds of developing a prolonged bTB herd breakdown. Co-infection with liver fluke may mask the true bTB infection status of animals making SICCT clearance of the herd difficult [18]. Indeed, given the widespread prevalence and high level of press coverage relating to fluke infection (and its potential link to bTB) it is surprising that not more herd keepers treat their cattle against liver fluke.

Other disease-related variables investigated were use of IBR (infectious bovine rhinotracheitis) vaccination and leptospirosis vaccination. Skuce *et al*. stated that the influence of respiratory infections on the susceptibility to infection with *M. bovis* remains untested but speculated that such infections can facilitate increased aerosol spread [12]. Our study showed an association between the use of IBR vaccination (used as a proxy for IBR exposure within a herd) and increased odds of developing a prolonged breakdown (OR 1.476). With leptospirosis vaccination, there was a negative association between its use and development of a prolonged bTB herd breakdown (OR 0.631), although the significance was marginal (*P* = 0.054) and given that 73% of these prolonged bTB herd breakdowns were in dairy herds may suggest that other management factors confound this finding.

The presence of registered pedigree animals on a farm was significantly associated with a reduced odds of developing a prolonged bTB herd breakdown. Association with this variable indicated that herds containing pedigree animals appear to be better at removing th infection. The presence of pedigree animals on a farm is probably indicative of a herd where trade and movement are important to the business, thus providing very strong motivation for a farmer to clear the infection from the herd and employ improved biosecurity measures.

Several studies have looked at the area of risk presented by slurry and manure derived from bTB-infected premises; but definitive results on the subject are few. Given the type of cases in our study and suggestions that approximately 6 months are required for deactivation of *M. bovis* in contaminated slurry [19], they must represent an extreme in terms of potential for the production of bTB-infected slurry or manure. The variable ‘manure spread on grazing ground’ had a positive association to prolonged bTB herd breakdowns (however not statistically significant in the final model, odds ratio = 1.289 (95% CI 0.926–1.798), a result consistent with previous work [19]), but was included as it provided adjustment effects for other variables. Our study indicated no association with the use of contractors for slurry spreading (at odds with another Northern Irish study [14]) or with applying slurry/manure to grazing ground. Even in situations where cattle can access ground to which fresh slurry has been applied, the results point to a negative association (OR 0.525) with cases. However, given the low number of cases (*n* = 17) in this category, it would require a guarded interpretation. The results show that herds located in the DVO north east group have the strongest statistical association to the prolonged breakdown case definition. Additionally, there is an interaction between DVO herd group and herd size (Fig. 2) where relative to the others, odds of a prolonged bTB herd breakdown increase for north east herds with increasing herd size.

Given that in this study, a statistical significance cut-off level of *P* < 0.05 was used for variable selection and that the final multivariable model contained 16 variables it should be realised that inclusion of at least one spurious association is a possibility. It is also possible with a study design where VOs select a breakdown source of infection there is potential for a degree of subjectivity. However, it is the trained VO with their local knowledge and standardised guidance who are best placed to make these assessments.

## Conclusions

One of the central tenets of this work was investigation of disease source and its relationship to long-duration bTB herd breakdowns. The source with the strongest association to long-duration breakdowns was purchase of infection; however, as a source, it applies mainly to beef fattening herds. Beef fattening herds mostly move their stock to an abattoir with very few cattle movements to other herds. However, they do present a risk from the continuous output of infection to local wildlife and to other herds grazed contiguously. In order to reduce the input of infection to these herds, they must have the capability to risk assess their purchases [20] thus reducing their overall ability to act as an infection focus in their locality. With herds other than beef fattening herds, the source of infection for long-duration breakdowns are multiple and must be addressed in a multi-faceted way.

In terms of wildlife source, more effort must be placed into breaking the transmission links between cattle and badgers. This could involve an array of methods varying from those directly applied to the badgers through to methodical and efficient fencing of badger setts and latrines. Indeed this work shows that basic segregation methods to separate badgers and cattle using fencing are not being applied. The low levels of fencing off by farmers could be as a result of confused communication, occurring where one section of government responsible for bTB control promotes it as necessary, while another section implementing subsidy payments (Basic Payment Scheme) contradicts it by officially removing these fenced off areas from field maps, potentially affecting payment. It is thus essential that governments do not create contradicting messages with policy implementation and should conceive more effective ways of promoting best practice [21], such as consulting widely before introducing future subsidies, to ensure their application is biosecurity friendly and communicating this effectively to the farming public. Indeed demonstrating what is possible, some areas fenced off as part of agri-environment schemes have now been deemed eligible for BPS area-based payments, a model which should be implemented in relation to farm biosecurity.

The other source shown to be linked to long-duration breakdowns was cattle to cattle contiguous herd spread and without effective biosecure boundaries between herds, this infection route will remain present. This again is an area where it should be possible for the government to intervene, incentivising the good practice of constructing biosecure boundary fences between neighbouring farms and penalising situations where poor fencing risks contiguous disease spread. Effective boundary fencing would form a necessary part of an overall biosecurity package aimed at the structural elements of the ongoing bTB problem.

## Data Availability

The data that support the findings of this study is not publically available data.

## Footnotes

1 R Core Team (2017). R: A language and environment for statistical computing. R Foundation for Statistical Computing, Vienna, Austria. URL https://www.R-project.org/.

2 StataCorp. 2017. Stata Statistical Software: Release 15. College Station, TX: StataCorp LP.

## Supplementary material

For supplementary material accompanying this paper visit http://dx.doi.org/10.1017/S0950268820002241. click here to view supplementary material
